# A Complete Hierarchical Key Management Scheme for Heterogeneous Wireless Sensor Networks

**DOI:** 10.1155/2014/816549

**Published:** 2014-04-10

**Authors:** Chien-Ming Chen, Xinying Zheng, Tsu-Yang Wu

**Affiliations:** ^1^School of Computer Science and Technology, Harbin Institute of Technology Shenzhen Graduate School, Shenzhen 518055, China; ^2^Shenzhen Key Laboratory of Internet Information Collaboration, Shenzhen 518055, China

## Abstract

Heterogeneous cluster-based wireless sensor networks (WSN) attracted increasing attention recently. Obviously, the clustering makes the entire networks hierarchical; thus, several kinds of keys are required for hierarchical network topology. However, most existing key management schemes for it place more emphasis on pairwise key management schemes or key predistribution schemes and neglect the property of hierarchy. In this paper, we propose a complete hierarchical key management scheme which only utilizes symmetric cryptographic algorithms and low cost operations for heterogeneous cluster-based WSN. Our scheme considers four kinds of keys, which are an individual key, a cluster key, a master key, and pairwise keys, for each sensor node. Finally, the analysis and experiments demonstrate that the proposed scheme is secure and efficient; thus, it is suitable for heterogeneous cluster-based WSN.

## 1. Introduction

Recently, wireless sensor networks (WSN) become more and more popular since they have been deployed in various applications, such as military, environmental monitor, industry automation, and smart space. A WSN is composed of a large number of sensor nodes which work together by collaborating with each other. In fact, sensor nodes are constrained in computing, communication, and energy capability; therefore, energy saving and hardware complexity are necessary to be considered carefully when constructing a WSN. For example, asymmetric cryptographic algorithms such as RSA or high cost operations like modular exponentiation operations are not appropriate for designing security mechanisms for a WSN.

Generally, all sensor nodes in a WSN may be divided into several small groups which are known as clusters [1–3]. Each cluster would have a cluster head responsible for collecting and aggregating sensing data from its cluster members. A cluster-based WSN can be implemented in both homogeneous WSN and heterogeneous WSN. We first consider the case that implementing a cluster-based WSN in homogeneous WSN. Note that all sensor nodes in homogeneous WSN have the same capabilities. After being deployed, every sensor node within the same cluster elects one as a cluster head. Obviously, the workload of acting a cluster head is heavier than a sensor node; as a result, the sensor node which acts as a cluster head would run out of its battery before other sensor nodes. Although it can be solved by rotating the role of the cluster head periodically over all cluster members, the workload of being a cluster head is still heavy for a sensor node. Actually, several studies [4, 5] have demonstrated that a homogeneous ad hoc network has poor performance and scalability.

Hence, several researches have concentrated on a heterogeneous WSN which incorporate different types of sensor nodes with different capabilities. For example, a WSN may contain a small number of powerful high-end sensor nodes (*H*-Sensors) and a large number of low-end sensor nodes (*L*-Sensors). If implementing a cluster-based WSN in heterogeneous WSN, *H*-Sensors organize *L*-Sensors around them into clusters, and *L*-Sensors forward sensing reports to the corresponding *H*-Sensor. It is commonly referred to as the heterogeneous cluster-based WSN. The advantage of it is the overall hardware cost of the entire WSN that can be reduced. This is because *L*-Sensors, which only perform the basic functions, can be manufactured very cheap and simple.

In another aspect, security is a vital issue in various WSN applications. Thus, an efficient key management scheme is necessary. Depending on different applications and security requirements, a variety of key management schemes have been proposed for a heterogeneous WSN [6–10]. In fact, most of these schemes place more emphasis on pairwise key establishment and key predistribution. However, due to the property of a heterogeneous cluster-based WSN that makes the topology hierarchical, a hierarchical key management scheme for cluster-based heterogeneous WSN is essential. Except for an pairwise key, which is shared between two sensor nodes, an individual key, which is shared between each sensor node and the base station, a common cluster key for each cluster, and a master key for all sensor nodes are also required.

Moreover, key updating is also required to be considered for the following reasons. First, sensor nodes may be compromised. The affected keys must be updated or revoked. Second, the network topology may be dynamic. For example, sensor nodes are deployed into the sea for aquatic application. If a sensor node moves to another neighboring cluster, some keys may need to be updated. Third, the base station may desire to update the master key periodically for better security level. For the best of our knowledge, no hierarchical key management scheme with key updating functionality for a heterogeneous cluster-based WSN has been proposed.

In this paper, we propose a complete and efficient hierarchical key management system for a heterogeneous cluster-based WSN. Our design only utilizes symmetric cryptographic algorithms and low cost operations such as bitwise XOR operation and modular multiplication. This construction contains two schemes, key generating scheme and key updating scheme. In the key generating scheme, four kinds of keys, which are an individual key, pairwise keys, a cluster key, and a master key, are generated for each *L*-Sensor. In the key updating scheme, we place the emphasis on updating cluster keys and the master key. In order to improve better efficiency, our design reduces the usage of unicasting. In the performance evaluation, we demonstrate that the storage requirement, communication, and computation cost are reasonable. Besides, in the security analysis, we show that the proposed construction is secure and influence of compromising can be confined to the affected cluster. Finally, the experimental results demonstrate that the operations used in our design are practical.

The remainder of this paper is organized as follows. In Section 2, some preliminaries are introduced. We describe the related work, network model, attack model, and our design goals. In Section 3, we propose our key generating scheme. In Section 4, the proposed key updating scheme is introduced. Then, Sections 5 and 6 describe the security analysis and performance evaluation of our design, respectively. We further provide experiments in Section 7. Finally, Section 8 concludes.

## 2. Preliminaries

In this section, the related work is introduced firstly. Then we describe the network model of heterogeneous cluster-based WSN and the attack model. We also list our design goals.

### 2.1. Related Work

Since several studies [4, 5] demonstrated that a homogeneous WSN has poor performance and scalability; several recent works investigate a heterogeneous WSN. Duarte-Melo and Liu [11] analyzed the energy consumption and lifetime of a heterogeneous WSN. Girod et al. [12] developed tools to support a heterogeneous WSN and measurement and visualization of operational systems. Du and Lin [13] proposed a differentiated coverage algorithm which can provide different coverage degrees for different areas. Lazos and Poovendran [14] studied the problem of coverage in planar heterogeneous WSNs. Lin et al. [15] proposed an ant colony optimization-based approach to maximize the lifetime of a heterogeneous WSN. Chen et al. [16] proposed a recoverable data aggregation scheme for a heterogeneous cluster-based WSN. As shown above, heterogeneous WSN indeed received lots of attention.

In the view of key management or key distribution schemes, several researches have been proposed [6–9, 17, 18]. Most of these schemes focus on probabilistic key predistribution method. Du et al. [6] presented an asymmetric key management scheme which preloads a large number of keys in each *H*-Sensor while preloading a small number of keys in *L*-Sensors. Later, Hussain et al. [8] also proposed key predistribution scheme, which reduces the storage requirements while maintaining the same security strength. Durresi et al. [7] proposed key predistribution schemes between stationary nodes and nonstationary nodes. Traynor et al. [9] described three keying and trust models for the heterogeneous WSN. Khan et al. [17] presented a key management scheme supporting mobility in a heterogeneous WSN that consists of mobile sensor nodes with few fixed sensor nodes. Shi et al. [18] proposed a resource-efficient authentic key establishment scheme for a heterogeneous WSN.

The above schemes for heterogeneous WSNs put more emphasis on pairwise key distribution and ignore the property of hierarchy. Thus, in this paper, we propose a hierarchical key management construction with the functionality of key updating.

### 2.2. The Network Model of Heterogeneous Cluster-Based WSN

The network model of a heterogeneous cluster-based WSN contains three components, a base station, a small number of powerful high-end sensor nodes (*H*-Sensors), and a large number of low-end sensor nodes (*L*-Sensors). *H*-Sensors are expected to have more energy than *L*-Sensors. They organize *L*-Sensors into clusters, collect and aggregate sensing data from their cluster members (*L*-Sensors), and send the results to the base station. Besides, an *H*-Sensor is equipped with a tamper-resistant hardware.

On the other hand, *L*-Sensors, which have sensing capability with limited computation, memory, and communication, are small and low-cost devices. Each *L*-Sensor detects a target within its detection range, uses its processing power to locally perform simple computations, and then sends the required data to the corresponding *H*-Sensor. Besides, an *L*-Sensor is not equipped with tamper-resistant hardware; therefore, an adversary can obtain the information stored in an *L*-Sensor after compromising it.

**Figure 1:**
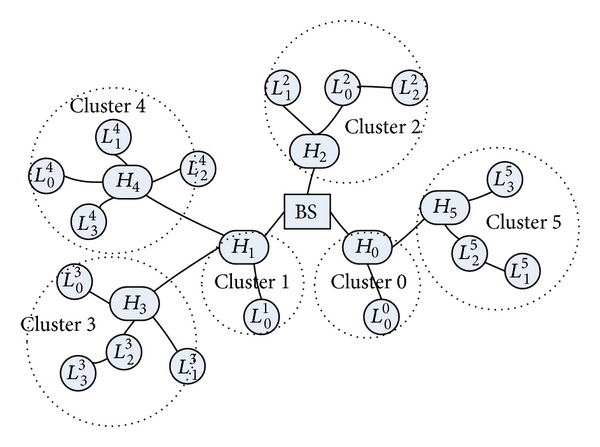
An example of a heterogeneous clustered-based WSN.

Figure 1 illustrates a simple example of a heterogeneous clustered-based WSN. Note that *H*_*i*_ denotes the *H*-Sensor *i* and *L*_*i*_^*j*^ denotes the *L*-Sensor; *i* belongs to *H*_*j*_. Depending on different environments, there might be more than one level of *H*-Sensors between the base station and *L*-Sensors. In Figure 1, cluster 3 contains *L*_0_^3^, *L*_1_^3^, *L*_2_^3^, *L*_3_^3^, and *H*_3_. *H*_1_, an upper level *H*-Sensor of *H*_3_, forwards the data sent from *H*_3_ to the base station.

Due to the properties of a heterogeneous WSN, the communication capacity of the base station, *H*-Sensors and *L*-Sensors are different. The communication can be classified into the following categories.

1. Within a cluster: an *H*-Sensor can broadcast/unicast messages to its cluster member through single hop, whereas messages sent to the *H*-Sensor may require multihop or still single hop depending on the distance between them.
2. Between two neighboring *H*-Sensors: an *H*-Sensor can communicate to neighboring *H*-Sensors through single hop.
3. Between the base station and *H*-Sensors: no doubt, messages sent to the base station require hop by hop. For example, in Figure 1, messages which are sent by *H*_3_ to the base station would pass through *H*_1_. On the other hand, the base station can broadcast/unicast to all *H*-Sensors through single hop or multihops.

### 2.3. Attack Models

Here we discuss the attack model for key management schemes in a WSN. Depending on the abilities of adversary, attacks can be categorized into two situations.

1. Without compromising sensor nodes: an adversary can only eavesdrop packets or send false messages to legal sensor nodes without any knowledge.
2. Compromising sensor nodes: after compromising a sensor node, an adversary can obtain the information stored in this sensor node. He may calculate other keys or secrets through the compromised information.

Note that detecting compromised sensor nodes which still act as normal sensor nodes is infeasible in all existing detection mechanisms in WSN. However, if an adversary compromises a sensor node and performs abnormal behavior or attacks, it will be detected [19–23]. Besides, in a heterogeneous WSN, it is assumed that every *H*-Sensor is equipped with a temper-resistant hardware; thus, considering the case that he compromises an *H*-Sensor is not required.

Based on the above attack models, a key management scheme for a WSN must satisfy the following security requirements.

 Requirement 1: all messages, including sensing data and control messages, must be encrypted.
 Requirement 2: an adversary cannot compromise the entire WSN with the compromised secrets. More specifically, he cannot derive the secrets that belong to other clusters. Furthermore, if he compromises two sensor nodes which belong to different clusters, he cannot obtain the secrets with other clusters either.
 Requirement 3: if compromised sensor nodes are detected, the affected keys must be updated or revoked.

### 2.4. Design Goals

Four kinds of keys, individual key, master key, cluster key, and pairwise key, are generated in the proposed construction.

1. Individual key: every *L*-Sensor and *H*-Sensor share an individual key with the base station. The base station can encrypt the secret information with the individual key if required.
2. Cluster key: every cluster has one cluster key which is shared with all cluster members including one *H*-Sensor and several *L*-Sensors. The cluster key is utilized for encrypting the cluster traffic. An *H*-Sensor can securely transfer control messages to its cluster members with this key. For example, an *H*-Sensor may turn some cluster members into sleep mode; it can encrypt this control message with the cluster key. On the other hand, all *L*-Sensors within the same cluster may encrypt the sensing reports with the same cluster key. The importance of cluster key is also discussed in [24].
3. Master key: this master key is shared between all *L*-Sensors and the base station within the entire network. The base station can securely broadcast the information to all *L*-Sensors with the master key.
4. Pairwise key: the pairwise key is shared with two neighboring *L*-Sensors. In some applications, two neighboring *L*-Sensors may require secure channel to protect their communication. For example, if the cluster key is compromised, *L*-Sensors in this cluster may encrypt the data with pairwise keys.

In this paper, we also consider how to update some of the above keys efficiently. Sensor nodes may be compromised or move to another neighboring cluster; consequently, the master key and the corresponding cluster keys must be updated. Besides, the master key may be updated periodically for security considerations.

## 3. The Proposed Key Generating Scheme

In this section, we propose a key generating scheme for a heterogeneous cluster-based WSN. As mentioned above, this scheme generates four kinds of keys, individual key, master key, cluster key, and pairwise key. Notations used in this paper are summarized as shown in Notation section.

### 3.1. Generating Each Individual Key

Actually, individual keys of each *L*-Sensor and *H*-Sensor are preloaded before being deployed. More specifically, an individual key *K*_*L*_*i*_^*j*^,BS_ is preloaded to *L*_*i*_^*j*^ and *K*_*H*_*j*_,BS_ is preloaded to *H*_*j*_.

After deployment, *H*-Sensors partition all *L*-Sensors into several clusters. Each *H*-Sensor can realize which *L*-Sensors are organized in its cluster. Then every *H*-Sensor reports its cluster information to BS.

Before generating other keys, BS requires to securely assign each *L*-Sensor a key which is shared with the attached *H*-Sensor. For example, *K*_*L*_*i*_^*j*^,*H*_*j*__ which is assigned to *L*_*i*_^*j*^ is shared between *L*_*i*_^*j*^ and *H*_*j*_. Since BS realizes the cluster information of each cluster, it can securely send the key *K*_*L*_*i*_^*j*^,*H*_*j*__ encrypted with *K*_*H*_*j*_,BS_ or *K*_*L*_*i*_^*j*^,BS_ to every *H*-Sensor and *L*-Sensor. Hence, all *L*-Sensors would share the keys with their attached *H*-Sensors. Note that the key *K*_*L*_*i*_^*j*^,*H*_*j*__ is transmitted only once before the stages of generating other kinds of keys.

### 3.2. Generating Cluster Keys

The procedure of generating cluster keys is modified from [25, 26]. To generate the cluster key, each *H*-Sensor constructs a binary tree and assigns its cluster members to leaf nodes. The root of the binary tree is the cluster key and intermediate nodes are key encryption keys. Note that the key encryption keys are used for updating the cluster key. Each *L*-Sensor knows all the keys from the parent of its corresponding leaf node up to the root. This set of keys is called the key path. The procedure of generating the cluster key is described as follows.

If there are *n*_*j*_ *L*-Sensors in cluster *j*, the *H*-Sensor *H*_*j*_ will generate random elements *R*_0_, *R*_1_,…, *R*_⌈log⁡_2_(*n*_*j*_)⌉−1_ and *K*_0_, *K*_1_,…, *K*_⌈*n*_*j*_/2⌉−1_ to calculate key encryption keys and the cluster key. Both key encryption keys and the cluster key are composed of one random element *R*_*X*_ and several random elements *K*_*Y*_, where 0 ≤ *X* ≤ ⌈log⁡_2_(*n*_*j*_)⌉ − 1 and 0 ≤ *Y* ≤ ⌈*n*_*j*_/2⌉ − 1. *R*_*X*_ means that this key is at level *X* in the key tree and *K*_*Y*_ means that this key belongs to the key path *Y*. All keys on the same key path *Y* have the same random elements *K*_*Y*_.

**Figure 2:**
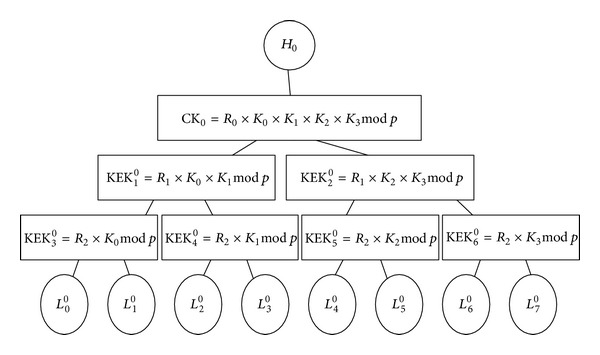
An example of a key tree.

The example in Figure 2 shows 8 *L*-Sensors in cluster 0. *H*_0_ constructs a key tree with 8 leaves and generates random elements *R*_0_, *R*_1_, *R*_2_, *K*_0_, *K*_1_, *K*_2_, and *K*_3_. In Figure 2, KEK_3_^0^ (=*R*_2_ × *K*_0_mod⁡⁡*p*), KEK_1_^0^ (=*R*_2_ × *K*_0_ × *K*_1_mod⁡⁡*p*), and CK_0_ (=*R*_0_ × *K*_1_ × *K*_2_ × *K*_3_mod⁡⁡*p*) are on the key path 0, KEK_4_^0^, KEK_1_^0^, and CK_0_ are on the key path 1, and so on. KEK_5_^0^, KEK_2_^0^, and CK_0_ belong to key path of *L*_4_^0^ and *L*_5_^0^. Note that *p* is a 128-bit prime number. Since CK_0_ is at level 0 in the key tree and belongs to the key path 0, 1, 2, and 3, it is composed of *R*_0_, *K*_0_, *K*_1_, *K*_2_, and *K*_3_. After the key tree is constructed, *H*_0_ assigns each cluster member *L*_*i*_^0^ to a leaf node and securely sends the cluster key CK_0_ and key encryption keys on the key path to *L*_*i*_^0^ using the key *K*_*L*_*i*_^0^,*H*_0__.

### 3.3. Generating the Master Key

Assume that there are *m* *H*-Sensors which are denoted as {*H*_0_, *H*_1_,…, *H*_*m*−1_} in a WSN. Before generating the master key, BS needs to deliver the secure information SI_*H*_*j*_,BS_ and SI_*L*_*i*_^*j*^,BS_ to each *H*-Sensor and *L*-Sensor. Note that this secure information is used when generating and updating other keys (see Section 4.2).

**Figure 3:**
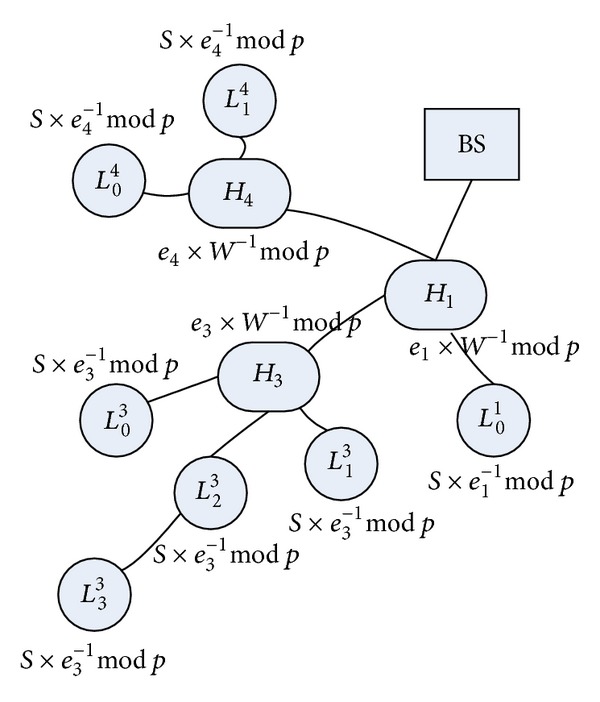
An example of the WSN with secure information.

BS first chooses a 128-bit prime number *p*, where *p* is public, and two secret random numbers *S*, *W* ∈ *Z*_p_*. It also selects *m* distinct numbers which are denoted as {*e*_0_, *e*_1_,…, *e*_*m*−1_} from *Z*_p_*. BS then securely sends the following message to all *H*-Sensors:

(1) $$\begin{array}{c} \forall 0\leq j<m,\;\text{BS}⟶H_{j}:E_{K_{H_{j},\text{BS}}}\left(\text{S}{\text{I}}_{H_{j},\text{BS}},\text{S}{\text{I}}_{L_{i}^{j},\text{BS}}\right), \end{array}$$

where SI_*H*_*j*_,BS_ = *e*_*j*_ × *W* ^−1^mod⁡⁡*p* and SI_*L*_*i*_^*j*^,BS_ = *S* × *e*_*j*_^−1^mod⁡⁡*p*. After receiving it, each *H*-Sensor, for example, *H*_*j*_, broadcasts *E*_CK_*j*__(SI_*L*_*i*_^*j*^,BS_) to all its cluster members. Note that CK_*j*_ is the cluster key of cluster *j*. As a result, all its cluster members can obtain SI_*L*_*i*_^*j*^,BS_. Note that *L*-Sensors attached to the same *H*-Sensor will have the same secure information SI_*L*_*i*_^*j*^,BS_.  Figure 3 shows an example of a WSN with secure information.

After that, BS starts to generate the master key. The procedure of master key generation is described as follows.

**Step 1**

BS first selects a random number *r* ∈ *Z*_p_* and computes a master key *K*_Master_ = *S* × *r*mod⁡⁡*p*.

**Step 2**

BS broadcasts *r* × *W*mod⁡⁡*p* to all *H*-Sensors.

**Step 3**

When each *H*-Sensor receives *r* × *W*mod⁡⁡*p* from BS, it calculates the following equations and broadcasts the result to all its cluster members.

For all 0 ≤ *j* ≤ *m* − 1, *H*_*j*_ calculates

(2) ((SIHj,BS)×(r×Wmod⁡⁡p)mod⁡⁡p)⊕CKj =((ej×W−1)×(r×Wmod⁡⁡p)mod⁡⁡p)⊕CKj =(r×ejmod⁡⁡p)⊕CKj.

We use bitewise XOR operation ⊕ to guarantee that messages can be securely sent to legitimate *L*-Sensors.

**Step 4**

When *L*_*i*_^*j*^ receives (*r* × *e*_*j*_mod⁡⁡*p*) ⊕ CK_*j*_ from *H*_*j*_, it computes the master key *K*_Master_, where

(3) KMaster=SILij,BS×(r×ejmod⁡⁡p)=(S×ej−1mod⁡⁡p)×(r×ejmod⁡⁡p)=S×rmod⁡⁡p.

By the above steps, all *L*-Sensors have the same master key *K*_Master_.

### 3.4. Generating Pairwise Keys

The pairwise key shared with two neighboring *L*-Sensors can be generated through their corresponding *H*-Sensor. For example, if *L*_0_^1^ desires to share a pairwise key with *L*_1_^1^, *H*_1_ will generate this pairwise key and send *E*_*K*_*L*_0_^1^,*H*_1___(pairwise_key) and *E*_*K*_*L*_1_^1^,*H*_1___(pairwise_key) to *L*_0_^1^ and *L*_1_^1^, respectively; consequently, both *L*_0_^1^ and *L*_1_^1^ can obtain this pairwise key.

## 4. The Proposed Key Updating Scheme

In this section, we propose the key updating scheme to update some of the generated keys. We discuss these kinds of keys separately as follows.

1. Individual key: since it is only shared between BS and each *L*-Sensor, this key is revoked automatically if an *L*-Sensor is compromised.
2. Cluster key: obviously, if an *L*-Sensor is compromised, the corresponding cluster requires updating the cluster key. Besides, *L*-Sensors may move to another neighboring clusters if the deployment of *L*-Sensors is nonstationary; consequently, both clusters (the original cluster and the target cluster) require updating their cluster key, respectively.
3. Master key: similarly, if *L*-Sensors are compromised, the master key must be updated. Besides, BS may desire to update the master key periodically for better security considerations.
4. Pairwise key: for example, assume that two *L*-Sensors, *L*_*i*_^*n*^ and *L*_*j*_^*n*^, have shared a pairwise key. If *L*_*i*_^*n*^ is compromised, *L*_*j*_^*n*^ would revoke the shared pairwise key automatically. Besides, these two *L*-Sensors can also update this key periodically.

### 4.1. Updating the Cluster Key

Here we discuss how to update cluster keys. First, we consider that an *L*-Sensor leaves a cluster. It is because this *L*-Sensor is compromised or moves to other neighboring cluster. Second, we consider an *L*-Sensor joins a cluster.

#### 4.1.1. An *L*-Sensor Leaves a Cluster

In Figure 2, if *L*_6_^0^ leaves cluster 0, the keys CK_0_, KEK_2_^0^, and KEK_6_^0^ must be updated. The detailed procedure is described in the following.

**Step 1**

*H*_0_ selects a random number *K*_3_′ from *Z*_p_* and sends the following key update messages:

- *H*_0_ → {*L*_0_^0^,…, *L*_3_^0^} : KEK_1_^0^ ⊕ ((KEK_1_^0^) × (*K*_3_^−1^ × *K*_3_′mod⁡⁡*p*)mod⁡⁡*p*),
- *H*_0_ → {*L*_4_^0^, *L*_5_^0^} : KEK_5_^0^ ⊕ ((KEK_5_^0^)×(*K*_3_^−1^ × *K*_3_′mod⁡⁡*p*)mod⁡⁡*p*),
- *H*_0_ → {*S*_7_^0^} : *K*_*L*_7_^0^,*H*_0__ ⊕  ((*K*_*L*_7_^0^,*H*_0__) × (*K*_3_^−1^ × *K*_3_′mod⁡⁡*p*)mod⁡⁡*p*).

**Step 2**

*S*_0_^0^, *S*_1_^0^, *S*_2_^0^, and *S*_3_^0^ can obtain *K*_3_^−1^ × *K*_3_′mod⁡⁡*p* with KEK_1_^0^ and then compute the new cluster key CK_0_′ where

(4) CK0′=CK0×(K3−1×K3′mod⁡⁡p)mod⁡⁡p=R0×K0×K1×K2×K3′mod⁡⁡p.

**Step 3**

*S*_4_^0^ and *S*_5_^0^ can obtain *K*_3_^−1^ × *K*_3_′mod⁡⁡*p* with KEK_5_^0^ and then compute the new keys CK_0_′ and KEK_2_^0′^ where

(5) KEK20′=KEK20×(K3−1×K3′mod⁡⁡p)mod⁡⁡p=R1×K2×K3′mod⁡⁡p.

**Step 4**

*S*_7_^0^ can obtain *K*_3_^−1^ × *K*_3_′mod⁡⁡*p* with *K*_*L*_7_^0^,*H*_0__ and then compute the new keys CK_0_′, KEK_2_^0′^, and KEK_6_^0′^ where

(6) KEK60′=KEK60×(K3−1×K3′mod⁡⁡p)mod⁡⁡p=R2×K3′mod⁡⁡p.

Obviously, it only requires updating the value *K*_3_ in this example. Since *L*_6_^0^ has no keys to obtain *K*_3_^−1^ × *K*_3_′mod⁡⁡*p*, it cannot compute the new cluster key and key encryption keys.

#### 4.1.2. An *L*-Sensor Joins a Cluster

When an *L*-Sensor joins a new cluster, the target *H*-Sensor authenticates this joined *L*-Sensor. This can be accomplished by coordinating with the original *H*-Sensor. Then BS would deliver a key which will be shared between the target *H*-Sensor and this *L*-Sensor. After receiving this key, the *H*-Sensor would assign this new *L*-Sensor a leaf node of the key tree. To prevent this new *L*-Sensor from decrypting the past traffic, all the keys on this key path need to be updated.

Let us take Figure 2 as an illustration. Assume that *L*_6_^0^ joins this cluster and has received an individual key *K*_*L*_6_^0^,*H*_0__. The procedure of updating the cluster key is described as follows.

**Step 1**

*H*_0_ assigns *L*_6_^0^ a leaf node of the key tree. *H*_0_ then selects a random number *K*_3_′ from *Z*_p_* to compute the new keys, CK_0_′, KEK_2_^0′^, and KEK_6_^0′^, where

(7)  CK0′=CK0×(K3−1×K3′)mod⁡⁡p=R0×K0×K1×K2×K3′mod⁡⁡p,

(8)  KEK20′=KEK20×(K3−1×K3′)mod⁡⁡p=R1×K2×K3′mod⁡p,

(9)  KEK60′=KEK60×(K3−1×K3′)mod⁡⁡p=R2×K3′mod⁡⁡p.

**Step 2**

*H*_0_ needs to send the following two messages; one is broadcasted to all *L*-Sensors within this cluster for updating the key path; one is additionally unicasted to the new *L*-Sensor *L*_6_^0^:

(i) *H*_0_ → {*L*_0_^0^,…, *L*_7_^0^} : CK_0_ ⊕ (*K*_3_^−1^ × *K*_3_′mod⁡*p*),
(ii) *H*_0_ → *L*_6_^0^:
  (10) (KL60,H0⊕(KL60,H0×CK0′mod⁡⁡p))||(KL60,H0⊕(KL60,H0×KEK20′mod⁡⁡p))||(KL60,H0⊕(KL60,H0×KEK60′mod⁡⁡p)).

Since *L*_6_^0^ does not have CK_0_, it cannot obtain *K*_3_^−1^ × *K*_3_′mod⁡⁡*p* to compute the previous keys. On the contrary, all other *L*-Sensors in this cluster will obtain *K*_3_^−1^ × *K*_3_′mod⁡⁡*p*.

**Step 3**

*L*_6_^0^ computes the new keys CK_0_′, KEK_2_^0′^, and KEK_6_^0′^ with *K*_*L*_6_^0^,*H*_0__.

**Step 4**

*L*_0_^0^, *L*_1_^0^, *L*_2_^0^, and *L*_3_^0^ can compute the new key CK_0_′ from (7) with the obtained *K*_3_^−1^ × *K*_3_′mod⁡⁡*p*.

**Step 5**

*L*_4_^0^ and *L*_5_^0^ can compute the new keys CK_0_′ and KEK_2_^0′^ from (7) and (8) with the obtained *K*_3_^−1^ × *K*_3_′mod⁡⁡*p*.

**Step 6**

*L*_7_^0^ can compute the new keys CK_0_′, KEK_2_^0′^, and KEK_6_^0′^ from (7), (8), and (9) with the obtained *K*_3_^−1^ × *K*_3_′mod⁡⁡*p*.

Similarly, only the value *K*_3_ is required to be updated.

#### 4.1.3. Further Discussion

Notice that in Step 1 of Section 4.1.1, for example, *L*_0_^0^ will receive *K*_3_^−1^ × *K*_3_′mod⁡⁡*p* protected by KEK_1_^0^. The reason that we use the equation

(11) $$\begin{array}{c} \text{KE}{\text{K}}_{1}^{0}⊕\left(\left(\text{KE}{\text{K}}_{1}^{0}\right)\times \left(K_{3}^{-1}\times K_{3}^{\prime }\mathrm{mod}p\right)\mathrm{mod}p\right) \end{array}$$

rather than

(12) $$\begin{array}{c} \left(\text{KE}{\text{K}}_{1}^{0}\right)\times \left(K_{3}^{-1}\times K_{3}^{\prime }\mathrm{mod}p\right)\mathrm{mod}p \end{array}$$

is to protect KEK_1_^0^. More specifically, if we only use (12), *L*_4_^0^, *L*_5_^9^, and *L*_7_^0^ can utilize the obtained *K*_3_^−1^ × *K*_3_′mod⁡⁡*p* to further obtain KEK_1_^0^. In fact, KEK_1_^0^ is not revealed to *L*_4_^0^, *L*_5_^0^, and *L*_7_^0^; therefore, an additional bitewise XOR operation ⊕ is required.

Similarly, in Step 2 of Section 4.1.2, the reason we use (10) rather than

(13) (KL60,H0×CK0′mod⁡⁡p)||(KL60,H0×KEK20′mod⁡⁡p)||(KL60,H0×KEK60′mod⁡⁡p)

is to protect *K*_*L*_6_^0^,*H*_0__; otherwise, other cluster members can obtain *K*_*L*_6_^0^,*H*_0__ with CK_0_′, KEK_2_^0′^, or KEK_6_^0′^.

### 4.2. Updating the Master Key

Here we discuss how to update the master key in the following situations. First, BS may update the master key periodically to improve the security level. Second, the master key is required to be updated if *L*-Sensors are compromised.

#### 4.2.1. Updating the Master Key Periodically

The master key may be updated periodically, for example, monthly. The procedure of updating master key is described as follows.

**Step 1**

BS selects a new random number *r*′ ∈ *Z*_p_* and calculates a new master key *K*_Master_new_ = *S* × *r*′mod⁡⁡*p*. BS broadcasts *r*′ × *W*mod⁡⁡*p* to all *H*-Sensors.

**Step 2**

While *H*_*j*_ receives *r*′ × *W*mod⁡⁡*p*, it calculates

(14) ((SIHj,BS)×(r′×Wmod⁡⁡p)mod⁡⁡p)⊕CKj =(r′×ejmod⁡⁡p)⊕CKj.

*H*_*j*_ then broadcasts the result to all its cluster members.

**Step 3**

*L*_*i*_^*j*^ can calculate the new master key *K*_Master_new_ where

(15) KMaster_new=SILij,BS×(r′×ejmod⁡⁡p)=(S×ej−1mod⁡⁡p)×(r′×ejmod⁡⁡p)=S×r′mod⁡⁡p.

In conclusion, all *L*-Sensors can obtain the new master key *K*_Master_new_.

#### 4.2.2. Updating the Master Key If *L*-Sensors Are Compromised

Assume that *L*-Sensor *L*_*m*_^*n*^ which is attached to *H*_*n*_ is compromised and *L*_*k*_^*n*^ denotes the *L*-Sensor *k* which is also attached to *H*_*n*_, where *k* ≠ *m*. Besides, there are *m* *H*-Sensors which are denoted as {*H*_0_, *H*_1_,…, *H*_*m*−1_} in the entire WSN. The procedure of updating the master key is described as follows.

**Step 1**

BS selects a new random number *r*′ ∈ *Z*_p_* and calculates a new master key *K*_Master_new_ = *S* × *r*′mod⁡⁡*p*. BS also selects a random number *e*_*n*_′ ∈ *Z*_p_* to compute *e*_*n*_^−1^ × *e*_*n*_′mod⁡⁡*p* and broadcasts ((*r*′ × *W*mod⁡⁡*p*)||(*E*_*K*_*H*_*n*_,BS__(*e*_*n*_^−1^ × *e*_*n*_′mod⁡⁡*p*))) to all *H*-Sensors.

**Step 2**

Except for *H*_*n*_, each *H*-Sensor calculates the following equations and broadcasts the result to its cluster members.

For all 0 ≤ *j* ≤ *m* − 1, *j* ≠ *n*, *H*_*j*_ calculates

(16) ((SIHj,BS)×(r′×Wmod⁡⁡p)mod⁡⁡p)⊕CKj =((ej×W−1)×(r′×Wmod⁡⁡p)mod⁡⁡p)⊕CKj =(r′×ejmod⁡⁡p)⊕CKj.

Then, all their cluster members can obtain the new master key *K*_Master_new_, where

(17) KMaster_new=SILij,BS×(r′×ejmod⁡⁡p)=S×r′mod⁡⁡p.

**Step 3**

(a) *H*_*n*_ obtains *e*_*n*_^−1^ × *e*_*n*_′mod⁡⁡*p* from *E*_*K*_*H*_*n*_,BS__(*e*_*n*_^−1^ × *e*_*n*_′mod⁡⁡*p*) and further computes the new secure information SI_*H*_*n*_,BS_′ where

(18) SIHn,BS′=(SIHn,BS)×(en−1×en′mod⁡⁡p)mod⁡⁡p=(en×W−1mod⁡⁡p)×(en−1×en′mod⁡⁡p)=en′×W−1mod⁡⁡p.

(b) *H*_*n*_ obtains (*r*′ × *e*_*n*_′mod⁡⁡*p*) ⊕ CK_*n*_ from *r*′ × *W*mod⁡⁡*p* and new secure information SI_*H*_*n*_,BS_′. Then *H*_*n*_ broadcasts ((*r*′ × *e*_*n*_′mod⁡⁡*p*)||(*e*_*n*_^−1^ × *e*_*n*_′mod⁡⁡*p*)) ⊕ CK_*n*_ ⊕ CK_*n*_′. Note that CK_*n*_′ is the new cluster key of cluster *n*.

(c) Except for *L*_*m*_^*n*^, all the other *L*-Sensors which are also attached to *H*_*n*_ can derive *e*_*n*_^−1^ × *e*_*n*_′mod⁡⁡*p* and *r*′ × *e*_*n*_′mod⁡⁡*p* from the obtained message ((*r*′ × *e*_*n*_′mod⁡⁡*p*)||(*e*_*n*_^−1^ × *e*_*n*_′mod⁡⁡*p*)) ⊕ CK_*n*_ ⊕ CK_*n*_′ and then compute SI_*L*_*k*_^*n*^,BS_′, where

(19) SILkn,BS′=SILkn,BS×(en−1×en′mod⁡⁡p)−1=(S×en−1mod⁡⁡p)×(en×en′−1mod⁡⁡p)=S×en′−1mod⁡⁡p.

(d) After updating SI_*L*_*k*_^*n*^,BS_′, all the other *L*-Sensors except *L*_*m*_^*n*^ within cluster *n* can compute new master key *K*_Master_new_ = *r*′ × *W*mod⁡⁡*p* where

(20) KMaster_new=SILkn,BS′×(r′×en′mod⁡⁡p)=(S×en′−1mod⁡⁡p)×(r′×en′mod⁡⁡p)=S×r′mod⁡⁡p.

Only *H*_*n*_ can obtain *e*_*n*_^−1^ × *e*_*n*_′ by decrypting *E*_*K*_*H*_*n*_,*BS*__(*e*_*n*_^−1^ × *e*_*n*_′mod⁡⁡*p*). Besides, the compromised *L*-Sensor *L*_*m*_^*n*^ cannot obtain *r*′ × *e*_*n*_′mod⁡⁡*p* and *e*_*n*_^−1^ × *e*_*n*_′mod⁡⁡*p* to compute the new secure information SI_*L*_*k*_^*n*^,BS_′ without CK_*n*_′; therefore, it is unable to obtain the new master key.

Obviously, only the *L*-Sensors within the affected cluster require updating the secure information before updating the master key; as a result, the influence can be confined locally.

#### 4.2.3. Further Discussion

We have already discussed how to update the master key. It may be questioned why we need to do it in this way. A trivial idea is to let BS generate a random master key (either for master key generation or master key updating). Then BS encrypts it using each cluster key and transmits it to every *H*-Sensor. This method seems simpler and more straightforward.

The reason is that the proposed scheme attempts to reduce the usage of unicasting. More precisely, in the above method, BS requires to unicast each encrypted master key to corresponding *H*-Sensor. However, BS only needs to broadcast the same message (*r*′ × *W*mod⁡⁡*p*) to every *H*-Sensor in our design. Actually, using broadcast is more efficient than using unicasting. We will demonstrate it through experiments in Section 7.

### 4.3. Updating the Pairwise Key

Assume that two *L*-Sensors, *L*_*i*_^*n*^ and *L*_*j*_^*n*^, have shared a pairwise key. If these two *L*-Sensors decide to update their shared pairwise key, *H*_*n*_ generates a new pairwise key and sends *E*_*K*_*L*_*i*_^*n*^,*H*_*n*___(new_pairwise_key) and *E*_*K*_*L*_*j*_^*n*^,*H*_*n*___(new_pairwise_key) to *L*_*i*_^*n*^ and *L*_*j*_^*n*^, respectively.

## 5. Security Analysis

In this section, we demonstrate that the proposed construction is secure through the following analyses. We first explain that our design satisfies the following security requirements mentioned in Section 2.3.

 Requirement 1: indeed, this requirement is satisfied. All kinds of messages (sensing reports and control messages) will be encrypted with the generated keys. An adversary cannot realize any information without keys.
 Requirement 2: if an adversary compromises an *L*-Sensor which is denoted as *L*_*i*_^*j*^, he can obtain *K*_master_ = *S* × *r*mod⁡⁡*p*, SI_*L*_*i*_^*j*^,BS_ = *S* × *e*_*j*_^−1^mod⁡⁡*p*, CK_*j*_, and several key encryption keys KEK. However, he is not capable of calculating cluster key and secure information belonging to other clusters. Moreover, the secrets belonging to other clusters cannot be derived even if he compromises two *L*-Sensors belonging to different clusters.
 Requirement 3: actually, the purpose of our key updating scheme can achieve Requirement 3.

Here we analyze the proposed construction in the following aspects.

### 5.1. The Influence of Compromised *L*-Sensors

Actually, an adversary can obtain all information which is stored in an *L*-Sensor after compromising it. Fortunately, the adversary cannot calculate the information (cluster key and secure information) of other clusters in our design; as a result, the proposed construction ensures that the compromise of *L*-Sensors does not cause the compromise of the entire network.

To prevent the adversary from decrypting future traffic, the compromised individual key and the pairwise keys are revoked automatically. Besides, BS updates the affected cluster key and the master key. In our design, the adversary cannot obtain this updated information. Moreover, as an example showed in Section 4, only the affected cluster needs to update the secure information before updating the master key. Obviously, the influence of compromising can be confined to be affected cluster.

Besides, if an *L*-Sensor moves to another cluster, both clusters (original and targeting clusters) would update the cluster keys. It can prevent the *L*-Sensor from decrypting the traffic of both clusters after it leaves or before it joins. In fact, it can also effectually reduce the influence of compromising. An adversary may eavesdrop and record all packets before compromising *L*-Sensors. Let us consider the following situations if an *L*-Sensor *L*_1_^0^ moves from cluster 0 to cluster 1. Note that CK_0_ and CK_1_ are the cluster keys of cluster 0 and cluster 1, respectively.

1. CK_0_ and CK_1_ are updated: CK_0_ and CK_1_ are updated to CK_0_′ and CK_1_′, separately. If *L*_1_^0^ which already has moved to cluster 1 is compromised, an adversary would obtain CK_1_′. Hence, he cannot decrypt the messages encrypted with CK_1_. Similarly, if the adversary compromises an *L*-Sensor within cluster 0 after *L*_1_^0^ leaves, he can only decrypt the messages encrypted with CK_0_′.
2. CK_0_ and CK_1_ are not updated: *L*_1_^0^ receives CK_1_ after arriving to cluster 1. If *L*_1_^0^ is compromised, an adversary can decrypt the messages encrypted with CK_1_. More specifically, after compromising *L*_0_^1^, he can retrieve the messages which are sent before *L*_1_^0^ joins. Similarly, the same condition happened if he compromises an *L*-Sensor within cluster 0.

Obviously, the influence of compromising is effectually reduced.

### 5.2. Confidentiality

An outsider cannot obtain the current cluster keys or master key because the cluster keys and the secure information *S* × *e*_*n*_^−1^mod⁡⁡*p* which is used for computing the master key are securely distributed to all legitimate *L*-Sensors. Although a leaving *L*-Sensor has the old secure information or the old cluster key, this *L*-Sensor cannot derive the new master key or the new cluster key. To prevent a leaving *L*-Sensor from obtaining the parameter *S* or *e*_*n*_, we choose the length of the prime number *p* as 128-bit. It is large enough to ensure that deriving correct (*x*,*y*) pair from *x* × *y*mod⁡⁡*p* is infeasible. Although a leaving *L*-Sensor knows the messages *r* × *W*mod⁡⁡*p* and *S* × *e*_*n*_^−1^mod⁡⁡*p*, it cannot compute the current master key *S* × *r*mod⁡⁡*p*.

### 5.3. Integrity

To achieve message authentication and integrity, we can utilize hash algorithms, for example, SHA-1 or MD5, to compute message authentication code (MAC) for a message. For example, in the master key generation phase of the key generating scheme, BS computes the MAC of *r* × *W*mod⁡⁡*p* with *K*_Master_, appends it to *r* × *W*mod⁡⁡*p*, and then broadcasts it to all *H*-Sensors. Each *H*-Sensor *H*_*j*_ broadcasts *r* × *e*_*j*_mod⁡⁡*p* and the received message (*r* × *W*mod⁡⁡*p* and its MAC) to its cluster members. Eventually, each *L*-Sensor uses the computed master key to recompute the MAC and compares it with the received MAC value. This implies that if an adversary masquerades as BS to send a message, he does not have the master key to compute the corresponding MAC value, and *L*-Sensors will reject this message.

## 6. Performance Evaluation

In this section, we show that the proposed construction is efficient in storage, communication, and computation. Besides, it is scalable because the additional overhead of increasing *L*-Sensors is confined to log_2_(*s*).

### 6.1. The Storage Requirement

In the key generating scheme, each *L*-Sensor requires storing some keys and necessary information. For example, an *L*-Sensor *L*_*i*_^*j*^, would store *K*_*L*_*i*_^*j*^,BS_, *K*_*L*_*i*_^*j*^,*H*_*j*__, SI_*L*_*i*_^*j*^,BS_, a cluster key CK_*j*_, and a common master key *K*_Master_. Also, several key encryption keys KEK must be stored to update the cluster key. The number of key encryption keys of *L*_*i*_^*j*^ is about ⌈log⁡_2_(*s*)⌉ − 1 where *s* is the number of *L*-Sensors in cluster *j*. The total storage of these secrets is 128-bit × (5 + (⌈log⁡_2_(*s*)⌉ − 1)). Since usually there are at most one hundred *L*-Sensors within a cluster, total storage is about 176 bytes. Comparing with the current generation of sensor nodes (128 Kbytes in programmable flash memory in MICAz), the storage of the proposed scheme is much less. Besides, as the cluster size grows, the number of keys stored in an *L*-Sensor increases proportional to the number of *L*-Sensors within the cluster in an order of log⁡_2_(*s*). Note that each *L*-Sensor may also require to store the pairwise keys shared with its neighbors if necessary, but the total storage requirement is still reasonable.

### 6.2. The Communication Cost

Here we discuss the communication cost of the proposed construction. The communication cost is closely related to two factors. The first one is the message size. Obviously, the number of bits of every message is less than 128-bit. It is reasonable in a WSN. The second one is the transmission types. In fact, using broadcasting is more efficient than using unicasting in a WSN (we will show it in the next section). The majority of transmission in the key generating scheme is using broadcasting. For example, in the master key generation phase, BS broadcasts *r* × *W*mod⁡⁡*p* to all *H*-Sensors. Similarly, *H*-Sensors also broadcast the calculating results to its cluster members. The use of unicasting in the proposed scheme is normally involved in the initial stage of some phases. For example, BS unicasts *E*_*K*_*H*_*j*_,BS__(SI_*H*_*j*_,BS_) to all *H*-Sensors at the initial stage of master key generation phase; thus, this transmission would be executed only once; even the master key must be updated. Similarly, transmitting *K*_*L*_*i*_^*j*^,*H*_*j*__ is still executed only once before generating cluster keys and the master key.

In the key updating scheme, every *L*-Sensor in the affected clusters only receives one message to update the cluster key. In order to avoid using unicasting, we aim to reduce the number of kinds of messages transmitted on the air. As the example shown in Section 4.1, only three kinds of messages are transmitted on the air. Similarly, only two kinds of messages are transmitted in Section 4.1. In the view of updating the master key, it only requires two broadcasts. More specifically, BS broadcasts same information to all *H*-Sensors, then each *H*-Sensor broadcasts the calculating result to all cluster members. Only the *L*-Sensors within the affected cluster require updating the secure information; consequently, the impact can be confined locally. As a result, updating keys incurs less communication overhead and is beneficial for the limited energy of *L*-Sensors.

### 6.3. The Computation Cost

The proposed construction utilizes symmetric cryptosystem, such as AES, and modular multiplication. AES is practical and efficient based on previous experimental studies. Another operation, modular multiplication, is always considered as an inefficient operation where the modulus is large, for example, 1024-bit moduli. However, the length of the modulus we adopted is only 128 bits. To demonstrate high effect of the modular multiplication with a 128-bit modulus, we implement it on MICAz sensor nodes. Computation results are given in Section 7.2.

## 7. Experiments

**Figure 4:**
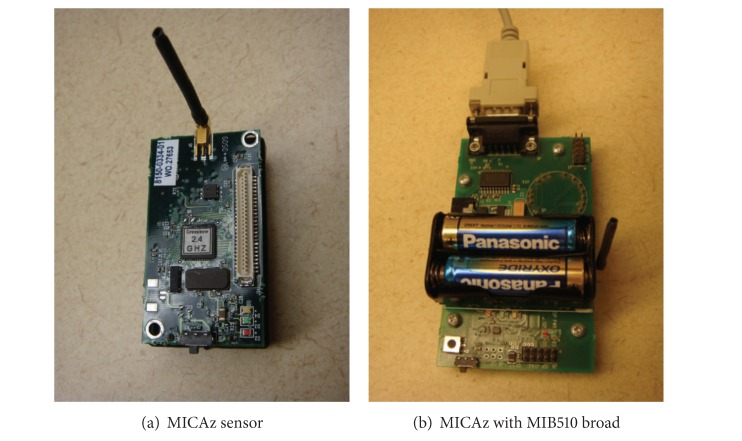
(a) Deployed sensor device and (b) base station device.

In our experiments, we choose MICAz sensor nodes. MICAz is capable of ATmega128L microcontroller. The architecture is 8-bit with 8 MHz computation speed. Total programmable memory storage of MICAz sensor is 128 Kbytes. For communication interface, MICAz uses ZigBee (802.15.4) to communicate with other MICAz sensors.  Figure 4(a) shows one MICAz sensor. Another device is the base station.  Figure 4(b) shows one MICAz sensor plugged on a MIB510 hardware interface, which is the interface of the base station. The MIB510 broad is connected to the desktop computer.

### 7.1. Experiment Assumptions and Design

**Figure 5:**
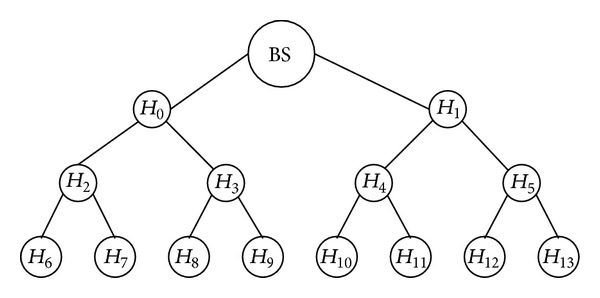
Example of a routing path where *d* = 2 and *h* = 4.

After deployment, routing paths will be constructed. Normally, a routing path is constructed as a tree structure. In order to simplify this experiment, we assume that all *H*-Sensors in the routing path have the same degree.  Figure 5 illustrates a constructed routing path with degree *d* = 2 and height *h* = 4.

Some researches assume that BS is capable of transmitting data to all *H*-Sensors through one hop. However, this assumption is not reasonable. In our experiments, we assume that data transmitted to all *H*-Sensors require multihops. For example, in Figure 5, the message sent to *H*_6_ by BS would pass through *H*_0_ and *H*_2_. Therefore, if BS desires to update the master key, the following two scenarios may happen using the example shown in Figure 5.

1. Using unicasting: BS encrypts the new master key *K* with each cluster key (CK_0_, CK_1_,…, CK_13_), respectively. BS then sends these encrypted master keys to all *H*-Sensors. Therefore, *H*_0_ receives seven messages which are *E*_CK_0__(*K*), *E*_CK_2__(*K*), *E*_CK_3__(*K*), *E*_CK_6__(*K*), *E*_CK_7__(*K*), *E*_CK_8__(*K*), and *E*_CK_9__(*K*). *H*_0_ obtains *E*_CK_0__(*K*) and then transmits three messages (*E*_CK_2__(*K*), *E*_CK_6__(*K*), and *E*_CK_7__(*K*)) to *H*_2_ and transmits the remainder three messages to *H*_3_. Similarly, *H*_4_ will receive three messages and then transmit two messages. Note that the size of the encrypted master key is 128 bits.
2. Using broadcasting: the proposed master key updating method actually uses broadcasting. BS broadcasts a common message to all *H*-Sensors. Every *H*-Sensor receives only one message from the upper level *H*-Sensor and then broadcasts it to lower level *H*-Sensors. For example, *H*_2_ receives a message from *H*_0_ and then broadcasts it to *H*_6_ and *H*_7_. Note that the size of the broadcasted message depends on two situations. First, if BS desires to update the master key periodically, the size of the broadcasted message is 128 bits (*r*′ × *W*mod⁡⁡*p*). Second, BS updates the master key if *L*-Sensors are compromised. The size of broadcasted message is 256 bits (((*r*′ × *W*mod⁡⁡*p*)||(*E*_*K*_*H*_*n*_,BS__(*e*_*n*_^−1^ × *e*_*n*_′mod⁡⁡*p*)))).

Three experiments are performed in this paper.

1. Experiment 1: we evaluate the energy consumption of an MICAz sensor node while transmitting or receiving messages with different sizes.
2. Experiment 2: we calculate the communication overhead of the entire network. We consider the above two scenarios.
3. Experiment 3: we evaluate the cost of AES and modulus multiplication.

In our experiments, we use MICAz sensor nodes to act as *H*-Sensors and calculate their energy consumption. If fact, energy consumed on powerful devices is the same as one on weak devices, such as MICAz. Besides, energy consumption measurement depends on the number of clock cycles spent [27]. Energy consumption for executing 2090 clock cycles on the ATmega128L microcontroller is equivalent to 7.4 *μ*J (Joule).

### 7.2. Experiment Results

**Experiment 1**

To measure energy consumed on transmitting and receiving data, the number of clock cycles is recorded when a data packet is received and sent. Broadcasting and unicasting executed 10 rounds for different length of data packets. The average results are showed in Table 1. Obviously, the values of broadcast 128-bit and unicast 128-bit are almost equal. Besides, the value of broadcasting 256-bit is larger but twice smaller than broadcasting 128-bit. It is because transmitting a 256-bit message still requires one package.

**Experiment 2**

According to the results in Experiment 1, total energy consumed for a WSN is simulated and evaluated.  Table 2 lists the result of communication overhead of the above two scenarios. We consider several routing paths with different degree *d* and height *h*. For example, if a routing path is generated as a tree with degree *d* = 2 and height *h* = 3, the total energy consumption of all *H*-Sensors when broadcasting 128-bit/broadcasting 256-bit/unicasting 128-bit is 0.0149/0.0174/0.3551 mJ, respectively. The number of *H*-Sensors in this tree is 6. While performing unicasting 128-bit, the maximum energy consumption among all *H*-Sensors is 0.0923 mJ. Besides, the average energy consumption of all *H*-Sensors is 0.0592 mJ when performing unicasting 128-bit.

Since senor nodes may be deployed in a large scale environment, the number of clusters may be up to thousands. Thus, we consider several candidates with different *d* and *h*. Obviously, the communication overhead is closely related to the number of *H*-Sensors. If it grows to hundreds or thousands of nodes, it causes huge energy consumption.

In the view of average energy consumption of an *H*-Sensor, we can consider the following cases.

1. Broadcast 128-bit: in this condition, every node receives one 128-bit message and broadcasts it. The total energy consumption of every node is 21.3 *μ*J (=14.2 + 7.1).
2. Broadcast 256-bit: similarly, the total energy consumption of every node is 24.84 *μ*J (=16.56 + 8.28).
3. Unicast 128-bit: the average energy consumption is listed in Table 2. Obviously, the values are quite larger.

**Experiment 3**

The goal of this experiment is to evaluate the costs of AES and modulus multiplication. Execution time and energy consumed by them are recorded. For AES, we choose an AES library based on TinyOS-2.x for comparison. We also implemented modulus multiplication by ourselves. These two operations were executed on MICAz physical sensors for 100 rounds. The average results are given in Table 3. In Table 3, one multiplication over 128-bit modulus is equivalent to 1.75 AES encryptions. As a result, modulus multiplication is feasible on physical sensors.

### 7.3. Discussion

Through these experiments, we can demonstrate that the proposed key updating scheme is efficient. This is because we utilize broadcasting instead of unicasting. Actually, we must take something into consideration. First, every *H*-Sensor in the routing path may not have the same degree. Second, BS may have more powerful transmission capability. Let us use Figure 5 as an example. The transmission range of BS may reach the second level *H*-Sensors. Therefore, the total energy consumption would not equal the result shown in Table 2. However, the overall energy consumption is still high if using unicasting.

## 8. Conclusion

In this paper, we proposed a complete hierarchical key management construction for heterogeneous cluster-based WSN which only utilizes simple operations. It considered several kinds of keys which are necessary for WSN. Besides, some kinds of keys may require updating; an efficient key updating scheme is also proposed. In order to provide better efficiency, the majority of transmission in our design is using broadcasting. In fact, using unicasting is inevitable in designing security mechanisms for WSN. Fortunately, the usage of unicasting in our design is normally involved at the initial stage of some phases. In the security analysis, we showed that the influence of compromising is effectually reduced and confined locally. We also showed that the proposed construction is efficient in storage, communication, and computation. Finally, we gave some experiments to further demonstrate two things. First, the operations we used are simple and practical. Second, using unicasting will cause uncontrollable overhead. In conclusion, the proposed construction is appropriate for heterogeneous cluster-based WSN.

## Figures and Tables

**Table 1:** Energy consumption of a MICAz sensor node.

	Transmit	Receive
Broadcast 128-bit	14.2 μJ	7.1 μJ
Broadcast 256-bit	16.56 μJ	8.28 μJ
Unicast 128-bit	14.1 μJ	7.05 μJ

**Table 2:** Communication overheads, unit is mJ.

Type	Broadcast	Broadcast	Unicast	Number of nodes	Maximum (uni)	Average (uni)
	128-bit	256-bit	128-bit			
*d* = 2, *h* = 2	0.0064	0.0075	0.0852	2	0.0355	0.0426
*d* = 2, *h* = 3	0.0149	0.0174	0.3551	6	0.0923	0.0592
*d* = 2, *h* = 4	0.0320	0.0373	1.1221	14	0.2059	0.0802
*d* = 2, *h* = 5	0.0660	0.0770	3.1105	30	0.4332	0.1037
*d* = 2, *h* = 6	0.1342	0.1565	7.9964	62	0.8877	0.1290
*d* = 2, *h* = 7	0.2706	0.3155	19.5862	126	1.7967	0.1554
*d* = 2, *h* = 8	0.5433	0.6336	46.4019	254	3.6147	0.1827
*d* = 2, *h* = 9	1.0887	1.2696	107.3052	510	7.2507	0.2104
*d* = 2, *h* = 10	2.1795	2.5417	243.6559	1022	14.5228	0.2384

*d* = 3, *h* = 2	0.0852	0.0994	0.2983	3	0.0852	0.0994
*d* = 3, *h* = 3	0.2769	0.3229	1.7896	12	0.2983	0.1491
*d* = 3, *h* = 4	0.8520	0.9936	8.1810	39	0.9374	0.2098
*d* = 3, *h* = 5	2.5773	3.0056	33.1077	120	2.8548	0.2759
*d* = 3, *h* = 6	7.7532	9.0418	125.1444	363	8.6071	0.3448
*d* = 3, *h* = 7	23.2809	27.1501	453.0253	1092	25.8640	0.4149

**Table 3:** Time and energy consumption for different operations.

	AES	Modular multiplication
Time (ms)	1.8	3.15
Clock cycle	1658.88	2903.04
Energy (μJ)	5.8735	10.2787

## Notation

BS: The base station
*H*_*j*_: The *H*-Sensor *j*
*L*_*i*_^*j*^: The *L*-Sensor *i* belongs to *H*_*j*_
*E*_*K*_(*M*): A message *M* encrypted with the key *K*
*K*_*L*_*i*_^*j*^,BS_: The predeployed key shared between *L*_*i*_^*j*^ and BS
*K*_*H*_*j*_,BS_: The predeployed key shared between *H*_*j*_ and BS
*K*_*L*_*i*_^*j*^,*H*_*j*__: The key shared between *L*_*i*_^*j*^ and *H*_*j*_
SI_*H*_*j*_,BS_: The secure information shared between *H*_*j*_ and BS
SI_*L*_*i*_^*j*^,BS_: The secure information shared between *L*_*i*_^*j*^ and BS
CK_*j*_: The cluster key for the cluster *j*
KEK_*l*_^*j*^: The key encryption key *l* in the cluster *j*
*K*_Master_: The master key
⊕: Bitewise XOR operation
||: Concatenation.
